# A 10-year experience of totally extraperitoneal endoscopic repair for adult inguinal hernia

**DOI:** 10.1007/s00595-014-1101-3

**Published:** 2015-01-07

**Authors:** Hiroki Toma, Toru Eguchi, Shuichi Toyoda, Yasuhiro Okabe, Tomonari Kobarai, Gen Naritomi, Takahiro Ogawa, Ichio Hirota

**Affiliations:** Department of Surgery, Harasanshin Hospital, Taihakucho, 1-10 Hakataku, Fukuoka, Japan

**Keywords:** Inguinal hernia, Laparoscopic surgery, TEP

## Abstract

**Purpose:**

Laparoscopic surgery is fast becoming the treatment of choice for inguinal hernia. By reviewing our 10-year experience of performing totally extraperitoneal repair (TEP), we sought to establish its clinical significance in the treatment of adult inguinal hernia.

**Methods:**

We reviewed retrospectively the clinical records of patients who underwent TEP for adult inguinal hernia between January 2003 and December 2012.

**Results:**

None of the 303 patients with adult primary or recurrent inguinal hernia in our study needed TEP converted to other procedures or suffered serious complications during the procedure. A significant difference was noted in the operation time between direct (*n* = 32) vs indirect (*n* = 128) hernias in the primary unilateral inguinal hernia group (91 ± 27 vs 80 ± 32 min, *p* = 0.033) and between direct/direct (*n* = 31) vs indirect/indirect (*n* = 24) hernias (136 ± 58 vs 89 ± 24 min, *p* = 0.01) in the primary bilateral inguinal hernia group. The only postoperative complications recorded were four cases of hernia recurrence (1.3 %) and one case of chronic pain (0.3 %).

**Conclusions:**

The results obtained for TEP over 10 years support this as a promising procedure for the treatment of adult inguinal hernia.

## Introduction

Laparoscopic surgery has become increasingly popular because patients want surgery that is minimally invasive. Hence, it has been gradually gaining acceptance as the treatment of choice for inguinal hernia. The main advantage of laparoscopic surgery for the treatment of adult inguinal hernia lies in the reduction of both the size of the wound and postoperative pain, thereby improving postoperative recovery as well as cosmesis [1–3]. Moreover, both the transabdominal preperitoneal approach (TAPP) and totally extraperitoneal repair (TEP) potentially allow the surgeon to avoid inguinal nerve injury that inevitably causes chronic pain, which compromises the patient’s quality of life [4–6]. The European Hernia Society (EHS) Guidelines recommend TEP rather than TAPP for laparoscopic approach, since TEP is performed outside the peritoneal cavity, contributing to a decreased incidence of the complications associated with the abdominal viscera such as visceral injury, port site hernia, and ileus [7]. Nonetheless, compared with the anterior approach, TEP is complicated and its mastery requires a long learning curve [8]. Consequently, the EHS Guidelines highlight the need for an expert to supervise TEP in clinical practice.

A recent randomized controlled trial comparing TEP with the Lichtenstein procedure demonstrated promising postoperative outcomes such as decreased postoperative pain and shorter recovery time for TEP [9], but practical data from a single institution seem insufficient. Thus, we report on our 10-year clinical experience of performing TEP for adult inguinal hernias from January, 2003 to December, 2012. Clinical records were reviewed retrospectively and short- and long-term outcomes were investigated. In this study, we sought to determine the clinical significance of TEP for the treatment of adult inguinal hernia.

## Materials and methods

The diagnosis of inguinal hernia was made based on a physical examination and computed tomography (CT) scans done with the patient in the prone position at the outpatient clinic. TEP was indicated in both primary and recurrent inguinal hernias, which were reducible in the supine position. TEP was contraindicated in cases of incarceration or after dissection of the preperitoneal space. All patients provided written informed consent before surgery. The clinical records of patients who underwent TEP for adult inguinal hernia between January, 2003 and December, 2012 were reviewed retrospectively. Clinical outcomes included the operation time, intra- and postoperative complications, length of postoperative hospital stay, hernia recurrence, and chronic pain (defined as pain that persisted for more than 2 months). Postoperative complications were graded according to the Clavien–Dindo Classification [10]. Patients were followed up in the outpatient clinic by the attending surgeons during the postoperative course. The follow-up period was defined as days from the operation to the latest consultation documented on the clinical records. Thus, the median follow-up period was 367 days for our series of patients. The statistical analysis for comparison of numerical data from two independent groups was performed by a two-tailed Student’s *t* test on SPSS ver. 8.01 (SPSS Inc, Chicago, IL). The nominal data from two independent groups were compared by the Chi-square test or Fisher’s exact probability test. Values are expressed as mean ± standard deviation (SD). A *p* value of less than 0.05 was considered significant.

### Procedure for TEP

A balloon-equipped trocar (Spacemaker Plus; Covidien, Mansfield, MA) was inserted into the preperitoneal space through a small infraumbilical incision and the balloon was inflated for 2 min to allow blunt dissection of the preperitoneal space. After deflating the balloon, pneumoperitoneum was created and maintained at 8 mmHg. Additional 2 ports were inserted in the midline of the lower abdomen, following which the preperitoneal space was dissected and the hernia sac was explored laparoscopically. In indirect inguinal hernia, the hernia sac was dissected from the spermatic sheath and divided after the ligation, whereas in direct inguinal hernia, the hernia sac was reduced immediately after the balloon dissection. The myopectineal orifice was repaired with inlay mesh that was secured with absorbable tacks (Absorba Tack; Covidien).

## Results

TEP was performed for adult inguinal hernia in 303 patients (Table 1). The primary inguinal hernias were classified using the categories outlined in Table 2. In the primary inguinal hernia group, the most frequent classification was indirect (73 %) in the unilateral group, whereas it was direct/direct (39 %) in the bilateral group. One unusual unilateral inguinal hernia with the hernia sac extending apart from the spermatic sheath towards the internal inguinal ring was unclassified. Five patients including one with spermatic cord lipoma had unclassified-type bilateral hernias.

**Table 1:** Patient characteristics

All patients (*n*)	303
Male (*n*)	287 (95 %)
Female (*n*)	16 (5 %)
Age at time of procedure (mean ± SD; years)	61 ± 13
Primary hernia (*n*)	279 (92 %)
Recurrent hernia (*n*)	24 (8 %)
Unilateral hernia (*n*)	202 (66 %)
Bilateral hernia (*n*)	101 (34 %)
Comorbidity (*n*)	Metabolic 33 (11 %)
	Cerebrovascular 12 (4 %)
	Cardiovascular 80 (26 %)
	Respiratory 5 (1.7 %)
	Digestive 22 (7.3 %)
	Urological 38 (12.5 %)
Anticoagulation therapy (*n*)	7 (2.3 %)

**Table 2:** Classification of primary inguinal hernia

Classification (*n*)	Unilateral (*n* = 186)	Bilateral (*n* = 93)
	Indirect 136 (73 %)	Direct/direct 36 (39 %)
	Direct 41 (22 %)	Indirect/indirect 28 (30 %)
	Femoral 1 (0.5 %)	Indirect/direct 12 (13 %)
	Pantaloon 7 (4 %)	Indirect/pantaloon 5 (5.4 %)
	Unclassified 1 (0.5 %)	Direct/pantaloon 3 (3.2 %)
		Direct/femoral 2 (2.2 %)
		Pantaloon/pantaloon 2 (2.2 %)
		Unclassified 5 (5 %)

**Fig. 1:**
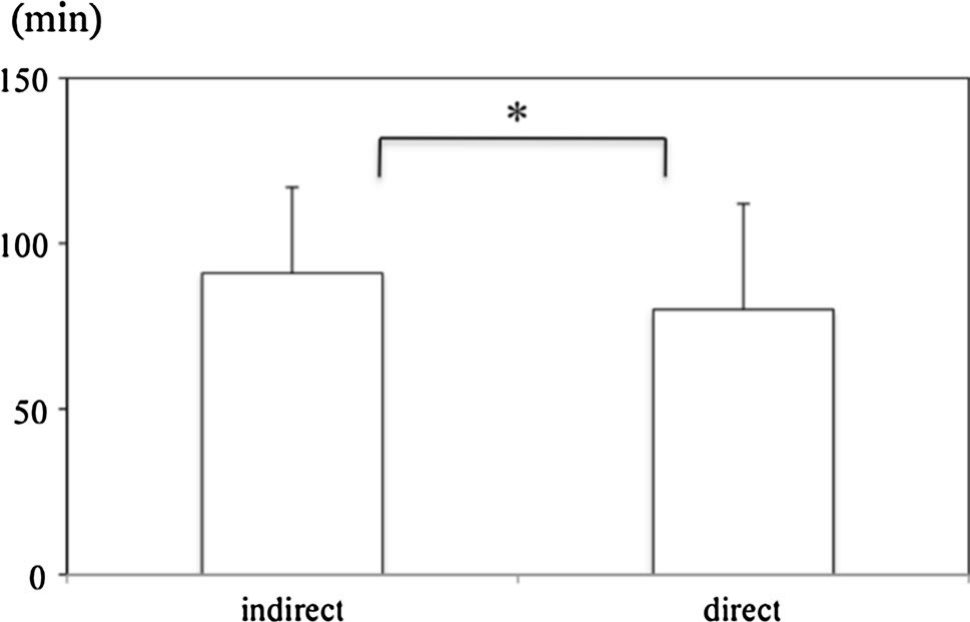
Operation time for primary unilateral indirect vs direct hernia repair. Values are expressed as mean ± SD. A significant difference was noted in the operation time between direct and indirect hernia repair (*p* = 0.033)

**Fig. 2:**
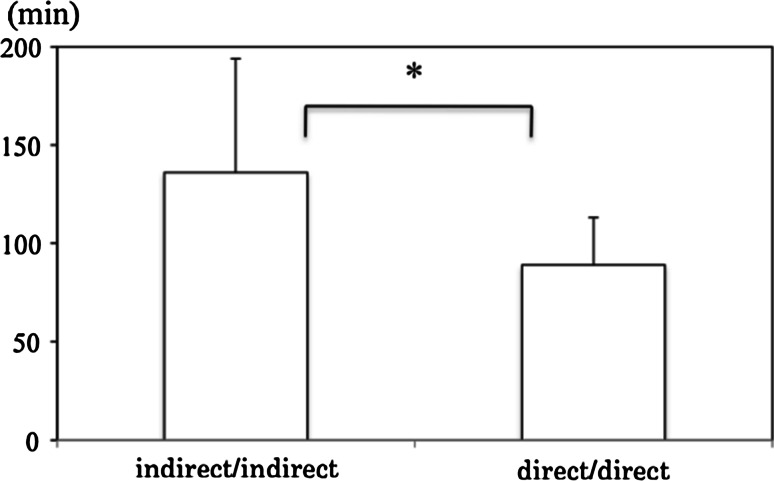
Operation time for primary bilateral indirect/indirect vs direct/direct hernia repair. Values are expressed as mean ± SD. A significant difference was noted in the operation time between indirect/indirect and direct/direct hernia repair (*p* = 0.01)

The operation time for primary inguinal hernia, excluding simultaneous surgery, was 89 ± 28 min in the unilateral group (*n* = 175) and 111 ± 44 min in the bilateral group (*n* = 86). There was a significant difference in the operation time for primary unilateral inguinal hernias between the direct (*n* = 32) and indirect (*n* = 128) groups (Fig. 1: 91 ± 27 vs 80 ± 32 min, *p* = 0.033). A similar trend was seen in the operation time for primary bilateral inguinal hernias between the direct/direct (*n* = 31) and indirect/indirect (*n* = 24) groups (Fig. 2: 136 ± 58 vs 89 ± 24 min, *p* = 0.01). There was no case of conversion to other procedures or serious complications during the operation. The peritoneal injury (*n* = 51) was repaired with an intracorporeal suture and the resulting pneumoperitoneum was deflated by the puncture with the needle placed into the peritoneal cavity during the operation. The postoperative hospital stay was 5 ± 4 days. Postoperative complications comprised four cases of hernia recurrence (1.3 %) and one case of chronic pain (0.3 %). All four cases of seroma and eight cases of subcutaneous hematoma, resolved with conservative treatment (Grade I in Clavien–Dindo Classification). In 24 patients with recurrent inguinal hernia after conventional non-mesh repair (*n* = 13), mesh-plug repair (*n* = 8), preperitoneal mesh repair (*n* = 3), TEP was completed without any serious intraoperative complications although postoperative subcutaneous hematoma (GradeI in Clavien–Dindo Classification) developed in two of these patients (Table 3).

**Table 3:** Intra-and postoperative complications (*n* = 303)

Intraoperative bleeding (*n*)	0
Visceral injury (*n*)	0
Conversion to other procedures (*n*)	0
Seroma (*n*)	4 (1.3 %)
Hematoma (*n*)	8 (2.6 %)
Recurrence (*n*)	4 (1.3 %)
Surgical site infection (*n*)	0
Chronic pain (*n*)	1 (0.3 %)
Others (*n*) ileus	2 (0.7 %)
Fever of unknown origin	1 (0.3 %)

## Discussion

Our review of clinical outcomes over 10 years clearly demonstrates the efficacy of TEP for the treatment of adult primary inguinal hernia. Moreover, the results of performing TEP to repair recurrent inguinal hernia were promising, but our data need further accumulation to establish the feasibility of using TEP for recurrent inguinal hernias. There was no case of conversion to other procedures or any intra- or postoperative serious complications. The rate of hernia recurrence was ~1.3 %, being relatively lower than that of previous reports [1, 2, 11]. In one of four patients with hernia recurrence, the recurrence developed on postoperative day 3 and was immediately repaired by repeat TEP. Another patient with hernia recurrence was treated with mesh-plug repair on postoperative day 309. In these two patients, the operative findings showed recurrence of an indirect inguinal hernia after the primary repair of an indirect inguinal hernia, suggesting that migration or shrinkage of the inlay mesh was the cause. The remaining two patients with hernia recurrence refused a re-operation.

During this 10-year period, 15 attending surgeons performed TEP under the supervision of an expert surgeon who had performed more than 500 TEP operations, as recommended by the EHS Guidelines. Zendejas et al. [12] reported the specific curriculum for surgical residents to improve their operative outcomes for TEP, demonstrating the effectiveness of simulation-based learning for the mastery of TEP. The establishment of a training system is essential for the promotion of TEP.

The magnified view obtained by laparoscopy helps the operator understand the anatomical structures, allowing for the precise diagnosis of the specific classification of inguinal hernia. In the primary inguinal hernia cases, we found a predominance of indirect unilateral hernias and direct/direct bilateral hernias, in accordance with previous reports [13, 14]. A significant difference in the operation time was noted between the direct and indirect primary unilateral inguinal hernia cases, presumably attributable to the difference in dissection of the hernia sac in TEP. The hernia sac is immediately reduced by balloon dissection in direct inguinal hernia but is consequently dissected from the spermatic sheath, resulting in a longer operation time for indirect inguinal hernia. There was a further significant reduction in operation time for the direct/direct type, compared with the indirect/indirect type of primary bilateral inguinal hernias. Therefore, a major advantage of TEP is its reduction of the operation time, since unilateral direct type hernia accounts for more than 60 % of primary bilateral inguinal hernia cases, as outlined in this study.

In analyzing the relationship of comorbidity to the occurrence of postoperative complications, there was no significant difference between comorbidity (metabolic, cerebrovascular, cardiovascular, respiratory, digestive, urological, anticoagulation therapy) and hematoma, seroma, and recurrence in the postoperative course (data not shown). One patient (0.3 %) suffered refractory inguinal pain that persisted for more than 2 months after TEP, and this was treated successfully with medication. Our data support the hypothesis that laparoscopic surgery results in a decreased incidence of postoperative chronic pain because of the reduced size of the wound, although it is known that various operative factors, including the choice of mesh (heavy vs light weight) and tacker (metallic vs absorbable vs non-fixation), also affect the duration of postoperative inguinal pain or discomfort [15–17]. According to a recent report, TEP resulted in more favorable postoperative outcomes than Lichtenstein procedure in relation to postoperative pain and faster return to daily activities [9], but our retrospective data were limited in revealing the superiority of laparoscopic surgery over the anterior approach for the treatment of adult inguinal hernia. Further accumulation of evidence is needed to establish the best surgical treatment for adult inguinal hernia.

In conclusion, a review of our 10-year experience of performing TEP demonstrated a lower incidence of intra- and postoperative complications, including hernia recurrence and chronic pain. These findings support TEP as a promising treatment for adult inguinal hernia repair.
